# The role of metabolic syndrome in sudden cardiac death risk: Recent evidence and future directions

**DOI:** 10.1111/eci.13693

**Published:** 2021-11-08

**Authors:** Amedeo Tirandi, Federico Carbone, Fabrizio Montecucco, Luca Liberale

**Affiliations:** ^1^ First Clinic of Internal Medicine Department of Internal Medicine University of Genoa Genoa Italy; ^2^ IRCCS Ospedale Policlinico San Martino Genoa – Italian Cardiovascular Network Genoa Italy; ^3^ Center for Molecular Cardiology University of Zürich Schlieren Switzerland

**Keywords:** dyslipidemia, hypertension, metabolic syndrome, sudden cardiac death

## Abstract

Metabolic syndrome (MetS) is a frequent condition whose deleterious effects on the cardiovascular system are often underestimated. MetS is nowadays considered a real pandemic with an estimated prevalence of 25% in general population. Individuals with MetS are at high risk of sudden cardiac death (SCD) as this condition accounts for 50% of all cardiac deaths in such a population. Of interest, recent studies demonstrated that individuals with MetS show 70% increased risk of SCD even without previous history of coronary heart disease (CHD). However, little is known about the interplay between the two conditions. MetS is a complex disease determined by genetic predisposition, unhealthy lifestyle and ageing with deleterious effects on different organs. MetS components trigger a systemic chronic low‐grade pro‐inflammatory state, associated with excess of sympathetic activity, cardiac hypertrophy, arrhythmias and atherosclerosis. Thus, MetS has an important burden on the cardiovascular system as demonstrated by both preclinical and clinical evidence. The aim of this review is to summarize recent evidence concerning the association between MetS and SCD, showing possible common aetiological processes, and to indicate prospective for future studies and therapeutic targets.

**Abbreviations**
BP: blood pressure
CAD: coronary artery disease
CHD: coronary heart disease
CMs: cardiomyocytes
CV: cardiovascular
CVD: cardiovascular disease
HDL‐C: high‐density lipoprotein cholesterol
HF: heart failure
HG: hyperglycaemia
HTN: hypertension
IFG: impaired fasting glucose
LDL‐C: low‐density lipoprotein cholesterol
LVH: left ventricular hypertrophy
MetS: metabolic syndrome
QTd: QT dispersion
SCD: sudden cardiac death
TGs: triglycerides
VAT: visceral adipose tissue
WC: waist circumference

## 1 INTRODUCTION

Metabolic syndrome (MetS) accounts for different metabolic disorders including impaired fasting glucose (IFG), high blood pressure (BP), abdominal obesity, high triglycerides (TGs) and low high‐density lipoprotein cholesterol (HDL‐C).^1^ From being a condition typically associated with Western lifestyle and habits, the incidence of MetS is now rising in developing countries with the result of becoming a real pandemic, urging rapid and worldwide counteractions. Indeed, to date, MetS is estimated to affect around one person every four with slightly increased prevalence in women.^2^, ^3^ Lifelong exposure to MetS components has been shown to exert deleterious effects in most systems and organs. Specifically, MetS deeply impacts on the cardiovascular (CV) system increasing CV risk by more than 2‐fold.^4^

Sudden cardiac death (SCD) is defined as a sudden unexpected arrest of CV system that occurs in a short time period (within 1 h of symptom onset) in subjects with or without previously recognized congenital or acquired cardiac alterations.^5^, ^6^ More than 50% of total cardiac death are defined as SCD.^7^, ^8^ In the United States, its incidence rates up to 60 per 100,000 persons every year, with men more likely of being victims of SCD as compared with women (rates: 76 and 45 per 100,000 persons, respectively).^9^

Though MetS is not included among risk factors for SCD, the relationship between its components and CV risk is well known.^4^, ^10^, ^11^ Recent studies evidenced a noteworthy increased risk—up to 70%^12^—of SCD among patients with MetS without history of coronary heart disease (CHD).^12^, ^13^, ^14^ In this review, we report on recent evidence associating MetS components with increased risk of SCD. Preclinical and clinical evidence will be dissected to summarize recent pathophysiological and clinical knowledge and highlight potential therapeutic target to reduce its burden.

## 2 METABOLIC SYNDROME: CLINICAL CRITERIA, RISK FACTORS AND PATHOPHYSIOLOGY

**FIGURE 1:**
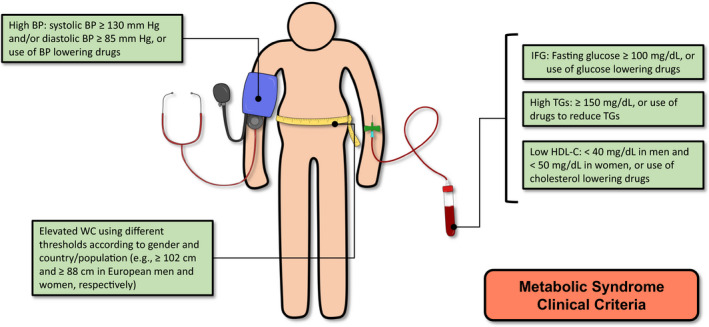
Metabolic syndrome diagnostic criteria. As reported by the International Diabetes Federation—American Heart Association/National Heart, Lung, and Blood Institute Joint Interim Statement definition.^15^ BP, blood pressure; HDL‐C, high‐density lipoprotein cholesterol; IFG, impaired fasting glucose; TGs, triglycerides; WC, waist circumference

MetS definition has slightly changed during the years.^1^ The clinical criteria of MetS according to the *International Diabetes Federation—American Heart Association/National Heart, Lung, and Blood Institute Joint Interim Statement* are summarized in Figure 1.^15^ The complex interplay among the different alterations characterizing MetS accounts for its high number of modifiable and nonmodifiable risk factors. Among the latter, genetic predisposition, male sex and ageing^3^, ^16^, ^17^ have been widely investigated with recent acquisitions highlighting the important role for genes related to lipid and glucose balance in MetS predisposition.^18^, ^19^ Among modifiable risk factors, unhealthy lifestyles—especially the association of overeating with reduced physical activity—favourite energy overflow, leading to adipose tissue hypertrophy. Adipose tissue not only has storage activity but can produce and release several molecules (i.e., adipokines) signalling at local and systemic levels. Adipokines are pivotal mediators of metabolic and CV pathophysiology and include pro‐inflammatory (i.e., tumour necrosis factor‐α, interleukin‐6, Leptin, Resistin, Lipocalin) and anti‐inflammatory (i.e., Adiponectin, secreted frizzled‐related protein 5) molecules.^20^, ^21^, ^22^, ^23^ The dysfunctional adipose tissue increases the synthesis and release of pro‐inflammatory adipocytokines, a condition known as metaflammation which is increasingly recognized as the common pathway of the different features of MetS.^24^ More specifically, MetS patients tend to have a lower adiponectin/leptin ratio that is considerable as an indicator of dysfunctional adipose tissue.^25^ Of interest, ageing—another MetS risk factor—contributes to the setting up of inflammatory and metabolic alterations in vulnerable individuals. Specifically, elderly people show chronically increased level of pro‐inflammatory mediators even in the absence of appropriate immunogenic stimuli (i.e., inflamm‐ageing) thought to facilitate different degenerative diseases including atherosclerosis and its ischaemic complications (ischaemic stroke and myocardial infarction). Metaflammation and inflamm‐ageing share different features and mechanisms that have been previously reviewed.^26^, ^27^

Obesity is classically associated with MetS^28^ beside not being essential for its diagnosis.^15^, ^29^ Indeed, recent evidence supports the fact that patients might be ‘metabolically obese’ even when showing normal weight and WC.^30^ In those patients, VAT within the intraperitoneal and retroperitoneal spaces is dysfunctional and associates with higher cardiometabolic risk.^31^, ^32^ VAT has an elevated lipolytic activity and can cause hyperinsulinaemia and insulin resistance.^33^ Furthermore, compared with subcutaneous adipose tissue, VAT shows increased inflammatory cell infiltration as well as metabolically active adipocytes.^34^ Consequently, the amount and functional status of VAT has been recognized as an important contributing factor to MetS.^35^, ^36^, ^37^, ^38^

Even though the precise mechanisms underlying MetS pathophysiology are not completely understood, insulin resistance and chronic inflammation are considered as the mainstays of MetS.^39^ Insulin resistance boosts adipose tissue lipolysis, blunts glucose uptake by muscles and causes an excess of hepatic production of glucose and TGs prompting higher circulating levels of free fatty acids and glucose.^39^ In people affected by MetS, hypertension (HTN) is probably related to renin‐angiotensin system activation and to the loss of insulin vasodilator effect on vessels.^40^, ^41^ Further, MetS patients seem to have increased levels of sympathetic nerve activity,^42^, ^43^, ^44^ causing reduced pancreatic insulin release, increased glucose production in the liver, augmented lipolysis in the adipose tissue and arterioles vasoconstriction.^42^ Moreover, since renin‐angiotensin system activity also depends on sympathetic nervous system, the higher sympathetic activity found in these patients could probably determine an angiotensin‐mediated increase in blood pressure values.^45^

## 3 SUDDEN CARDIAC DEATH AND METABOLIC SYNDROME

Many diseases are associated with SCD such as diabetes,^46^ HTN,^47^ left ventricular hypertrophy (LVH),^48^ CHD^49^ and heart failure (HF),^49^ but the strength of these associations vary among different populations, age and sex groups. Coronary artery disease (CAD) is listed as the most important cause of SCD,^50^, ^51^ being causative for almost 75% of total SCD burden in Western countries.^52^ Also, genetic cardiac abnormalities are important causes of SCD especially in subjects aged less than 35 years, whereas CAD represents the leading underlying cause in victims aged more than 35 years.^52^

The pathogenesis of SCD highly depends on its causative factor, although common final pathways are thought to sustain the final events. Specifically, the most accepted mechanism is the onset of a ventricular arrhythmia, caused by different possible triggers, such as acute ischaemia, ion disorders or structural alterations.^52^ Ventricular tachyarrhythmias are frequent and often lethal, estimated to sustain up to 80% of total SCD.^7^ They show circadian and infradian variations (i.e., higher prevalence of SCD during winter and in the morning) and can be triggered by vigorous physical activity and psychological stressors such as anxiety and depression).^52^ As previously mentioned, in most cases, an acquired or genetic cardiac disorder constitute the background for such fatal arrhythmias.^53^

As for MetS, the sympathetic nervous system could play an important role in determining SCD as well.^54^ In younger subjects, ventricular arrhythmias seem to be associated with an excess of heart sympathetic stimulation, particularly when the arrhythmic risk is genetically determined.^55^ Specifically, sympathetic activation plays an important role in subjects with long QT and catecholaminergic polymorphic ventricular tachycardia.^55^, ^56^ On the other hand, the sympathetic system may exert a protective effect in patients with Brugada or J‐wave syndromes.^57^ Less is known on its role in older subjects including those with MetS. However, a higher sympathetic activity has been involved in the formation of atherosclerotic plaque and can cause coronary vasoconstriction,^58^, ^59^ therefore favouring cardiac ischaemia. Furthermore, some evidence also supports a role for sympathetic triggering of arrhythmia in elderly patients with CHD.^57^ However, the extent of such an association remains to be fully disclosed.

## 4 HYPERGLYCAEMIA

According to the American Diabetes Association, IFG is defined as the presence of fasting plasma glucose concentrations between 100 and 125 mg/dl (5.6 to 6.9 mmol/L) and impaired glucose tolerance as plasma glucose concentrations between 140 and 199 mg/dl (7.8 to 11.0 mmol/L) at the second hour of the oral glucose tolerance test.^60^ Patients with IFG, impaired glucose tolerance or haemoglobin A_1c_ between 5.7 and 6.4% are at high risk of developing overt diabetes,^60^ such conditions are often defined as prediabetes.^61^ IFG, impaired glucose tolerance and diabetes alongside their interplay with the other components of MetS have direct and indirect deleterious effect on cardiac function and predispose to SCD as demonstrated by several basic science as well as clinical evidence.

### 4.1 Basic science research

Hyperglycaemia (HG) has been shown to have different damaging effects on cardiomyocyte (CM) function and survival. Exposure to high glucose environment induces CM death both in vitro and in vivo in different animal models of disease including ischaemic^62^, ^63^, ^64^, ^65^ and non‐ischaemic ones.^66^, ^67^, ^68^, ^69^ Lower levels of adenosine triphosphate (ATP), adenosine monophosphate (AMP), fumarate, succinate, aspartate and creatinine, alongside increased branched‐chain amino acids content in CM exposed to HG favour their apoptosis.^69^ Similarly, also oxidative stress through its activating effect on cytochrome c‐activated caspase‐3 pathway can explain the higher rate of apoptosis in CMs exposed to HG conditions.^66^

Moreover, HG was shown to affect the contractile function of CMs by impairing intracellular calcium balance and causing ventricular dilatation and systolic disfunction.^70^ Also, HG can prolong action potentials of CMs by inducing calcium mismatch.^71^ In rat models of prediabetes fed with a high‐sucrose diet, HG was shown to induce LVH, independently from obesity and HTN.^72^ Furthermore, in streptozotocin‐induced diabetic rats, HG causes QT interval elongation, with cardiac repolarization being reversed only after 4 days of insulin infusion.^73^

HG favours the atherosclerotic process in different ways.^74^, ^75^ An interesting explanation of this complex process concerns the formation of toxic products after the interaction between glucose and other molecules. More specifically, advanced glycosylation end products are formed because of the interaction between proteins, lipid or nucleic acids and glucose via chemical processes of non‐enzymatic glycation and oxidation.^76^ In general, the more blood glucose is present, the more advanced glycosylation end products are likely to be created.^77^ Advanced glycosylation end products have been shown to play important role in regulation of inflammation, extracellular matrix modifications, lipoprotein modification and cellular proliferation, all processes deeply involved in the pathophysiology of atherosclerosis.^74^, ^78^

### 4.2 Clinical research

Preclinical evidence found confirmation in clinical cohorts as diabetes and prediabetes individuals show increased all‐cause and CV mortality.^79^ As reported by a recent meta‐analysis, those individuals have a 2‐fold higher risk and a 23% of increased risk, respectively, of SCD compared with people without glucose alterations.^80^ In a prospective study assessing middle‐aged men, non‐diabetic subjects with IFG showed a 1.5‐fold risk of SCD compared with patients showing normal fasting plasma glucose even after adjustments for other CVDs risk factors.^81^ Remarkably, the authors showed that every 1 mmol/L augmentation of fasting plasma glucose was associated with a 10% increase of SCD risk.^81^ Moreover, haemoglobin A_1c_ is associated with SCD risk, even among patients without previously known CVDs.^82^ Yet, the association between diabetes and SCD is far from being non‐controversial, with a recent report based on competing risk analysis highlighting that in primary prevention implantable cardiac defibrillator (ICD) patients, diabetes is associated with the risk of death without, but not with, sustained ventricular arrhythmias and appropriate ICD therapies.^83^ Of importance, diabetes mellitus is associated with several causes of sudden cardiac arrest including CHD, HF with preserved or reduced ejection fraction, and arrhythmias particularly due to QT prolongation.^84^ Thus, a prospective population‐based cohort study evidenced that IFG predispose to QT elongation and reduction of RR interval.^85^ Also, the authors demonstrated a higher risk of SCD among these patients and proposed sympathetic activity as the main cause of RR shortening.^85^

As long‐time recognized, diabetes and prediabetic conditions are among the more prevalent causes of CHD. Several studies showed a higher risk of CAD in patients with or without diabetes in correlation with their glycaemic profile.^86^, ^87^, ^88^, ^89^, ^90^ However, CAD prevalence is higher among diabetic subjects rather than non‐diabetic ones.^89^ Also, a diet with elevated glycaemic load is related to CAD.^91^, ^92^ Elevated fasting plasma glucose is independently predictive of LVH in patients without diabetes even without prior history of CVDs.^93^, ^94^ Prediabetes and diabetes associate with higher risk of HF^95^, ^96^, ^97^, ^98^, ^99^ by increasing the risk of CAD, heart microvascular function and LVH.^95^ Furthermore, insulin resistance is associated with a higher risk of diastolic dysfunction in both prediabetes^100^ and diabetic^101^, ^102^ patients. Such an association is probably due to the reduced glucose uptake found in cardiomyocytes in cases of insulin resistance, leading to metabolic alterations that pave the way towards chronic HF.^103^

## 5 HYPERTENSION

According to the 2018 ESC/ESH Guidelines,^104^ HTN is defined as the presence of elevated systolic and/or diastolic BP (≥140 and ≥90 mm Hg, respectively). With respect to MetS diagnosis, the Joint Interim Statement includes individuals with high normal BP (≥130 systolic and/or diastolic ≥85 mm Hg) or those taking anti‐HTN medications. HTN accounts for an important part of the total burden of diseases^15^; as an example, a recent study in Italy quantified a prevalence of hypertension up to 26% in MetS individuals.^105^

### 5.1 Basic science research

Increased blood pressure values exert several detrimental effects on the heart. Mechanical straining, oxidative stress, neurohormonal activation (i.e., renin‐angiotensin and sympathetic system activation) and upregulation of pro‐inflammatory cytokines account for the increased CM apoptosis observed in basic research studies with hypertensive models.^106^ Increased levels of norepinephrine cause CM apoptosis in neonatal rats in a time‐ and dose‐dependent manner,^107^ while increased CM apoptosis favourites the transition from LVH to overt ventricular dysfunction in different animal models of HTN.^108^, ^109^

Hypertensive animal models have a higher risk of developing cardiac hypertrophy.^108^, ^110^, ^111^, ^112^, ^113^ Rat hearts working against pressure overload show a higher risk of metabolic impairments, such as an augmented glucose uptake in CMs and LVH.^114^ Such metabolic alterations precede the onset of overt cardiac hypertrophy or ventricular dysfunction^114^ and may partially account for the episodes of SCD reported in hypertensive male rats.^115^ Accordingly, LVH associates with SCD in patients.^116^, ^117^, ^118^

Nevertheless, hypertension and LVH are underlined by complex intracellular alterations that may account themselves for the risk of SCD. For instance, mice overexpressing angiotensin‐converting enzyme show a higher risk of arrhythmias and SCD even when having normal blood pressure and no ventricular hypertrophy.^119^ On the other hand, hearts of normotensive mice exposed to high levels of angiotensin II are more prone to develop cardiac hypertrophy^120^ and ventricular dysfunction,^121^ while blocking angiotensin receptors may reduce arrhythmias in LVH mice.^122^ High levels of oxidative stress, together with increased inflammation, ion transport alterations and morphological changes have been identified as possible underlying mechanisms of the association between hypertension, LVH, arrhythmias and SCD.^123^, ^124^, ^125^, ^126^

### 5.2 Clinical research

Hypertension is a known risk factor for most causes of SCD. Accordingly, men with basal systolic BP >145 mm Hg as compared with normotensive patients have a 2‐fold increased risk of SCD^127^ and every 20/10 mm Hg increase of systolic/diastolic BP associates with up to 20% higher risk of SCD.^128^ HTN causes LVH and cardiac hypertrophy is associated with a higher risk of SCD,^117^, ^118^, ^129^ especially in black people.^52^, ^130^ Even though the pathogenesis of this association is not fully understood, LVH not only alters the cardiac muscular structure but also its electric activity and hypertrophic hearts have a higher risk of arrhythmia onset.^118^, ^131^, ^132^ Indeed, LVH favours cardiac fibrosis in hypertensive subjects,^133^ and cardiac fibrosis impairs heart conduction, eventually favouring arrhythmias.^134^, ^135^, ^136^

Cardiac hypertrophy initially compensates the hypertensive state, but this adaptation becomes pathological when the hypertensive stimulus reiterates during the time. LVH is common among subjects affected by HTN, but even people with high normal blood pressure have a higher risk of developing LVH compared with normotensive subjects.^137^ Recently, genetic factors have shown an important role in developing LVH as a response to BP increase.^138^ In this study, every 10 mm Hg augmentation of genetically predicted systolic BP was associated with almost 5 g of left ventricular mass increment.^138^

However, some studies reported that the ventricular dysrhythmias found in hypertensive subjects with LVH may have weak associations with SCD.^139^, ^140^ Nonetheless, the presence of cardiac or non‐cardiac comorbidities increases the risk of SCD in patients with LVH. Among them, the presence of cardiac ischaemia or fibrosis,^48^ but also renal failure requiring haemodialysis which was reported to increase the sympathetic activity and the risk of SCD in patients with LVH.^141^

## 6 WAIST CIRCUMFERENCE, TRIGLYCERIDES AND HIGH‐DENSITY LIPOPROTEINS

WC is used to assess abdominal fat. Even though imaging techniques are needed to precisely estimate the amount of VAT, WC correlates with VAT irrespective of ethnicity and gender differences.^142^, ^143^

High TGs and low HDL‐C blood levels takes part to MetS diagnostic criteria.^15^ HDL particles transport cholesterol from the periphery to the liver,^144^ whereas TGs are energetic molecules used by cells via β‐oxidation or stored in adipocytes as stocks.^145^ Lack of HDL‐C and excess in TGs are related to a higher risk of CHD, especially in women.^146^ In MetS subjects, the excess of free fatty acids in the blood enhances gluconeogenesis, lipogenesis, reducing glucose sensitivity and favouring the onset of further metabolic disturbances.^39^

### 6.1 Basic science research

Up to 70% of cardiac adenosine triphosphate derives from fatty acid β‐oxidation,^147^ and obesity is associated with an increased cardiac fatty acid β‐oxidation.^148^ Accordingly, mouse models of obesity show increased TGs uptake in CMs.^148^, ^149^ However, TGs—when in excess—may cause detrimental effects on the heart. This condition is known under the name of cardiac ‘lipotoxicity’ and includes reactive oxygen species production, release of mitochondrial cytochrome c and apoptosis, as the main mechanisms of damage.^150^, ^151^ Accordingly, a study assessing mice with carnitine palmitoyltransferase‐1b deficiency—a protein involved in β‐oxidation—showed that these mice have a higher TGs accumulation in the myocardium, leading to cardiac hypertrophy, impaired cardiac contractions, congestive HF and premature death after cardiac pressure overload when compared with control mice.^152^ Similarly, mice with deficiency of very‐long‐chain acyl‐coenzyme A dehydrogenase, another crucial enzyme of β‐oxidation, that are fed with a diet rich of TGs show a noteworthy increment of VAT, resulting in the development of MetS‐like phenotype.^153^

As for arrhythmias, evidence suggests that high‐fat diet can trigger ventricular arrhythmias in mice through the release of Ca^++^ from sarcoplasmic reticulum and ion channel activation.^154^ Similarly, another study demonstrated the presence of QT intervals elongation, QT dispersion (QTd) augmentation, increased sympathetic innervation, an augmented Ca^++^ current density and a higher risk of ventricular fibrillation in the heart of rabbits fed with a high cholesterol diet.^155^ Of interest, administration of simvastatin reduced the risk of ventricular fibrillation in similarly fed rabbits.^156^

### 6.2 Clinical research

Victims of SCD are likely to have a wider WC,^13^ though the correlation between abnormal WC and SCD is not completely demonstrated now. In fact, a recent meta‐analysis showed that both the augmentation of waist‐to‐hip ratio and BMI were predictive of SCD risk, whereas no dose‐response conclusions about WC were possible because of too few studies including this parameter.^157^

Nonetheless, WC is associated with electrocardiographic alterations that may predispose the onset of arrhythmias. For instance, a higher WC correlates with prolonged QTc interval in obese young men.^158^ Also, the QRS|T angle is frequently abnormally deviated in MetS individuals, particularly among MetS women elevated abdominal obesity.^159^ Of interest, the QRS|T angle represents the difference between cardiac depolarization and repolarization, and its alteration has been related to ventricular arrhythmias and therefore prediction of SCD.^160^

Dyslipidaemia can concur in causing SCD through the development of coronary atherosclerosis and acute ischaemic CV events.^161^ HDL‐C protects against atherosclerotic plaques by favouring reverse cholesterol transport and through its anti‐oxidant and anti‐inflammatory properties.^162^, ^163^ Indeed, TGs/HDL‐C^164^ and low‐density lipoprotein cholesterol (LDL‐C)/HDL‐C ratios^165^ are directly related with SCD risk. Notably, low HDL‐C levels are also an independent predictor of left ventricular mass in both treated and untreated hypertensive patients,^166^, ^167^ whereas TGs showed a weak association.^166^, ^167^ Similarly, elevated plasma TGs play a role in the development of atherosclerosis.^168^, ^169^, ^170^ Accordingly, patients with ‘hypertriglyceridemic waist’, which consists of both abnormal TGs levels and elevated WC, have a higher risk of CAD (20643837) and higher amount of visceral adipose tissue.^171^

## 7 METABOLIC SYNDROME AND SUDDEN CARDIAC DEATH INTERRELATIONS AND FUTURE DIRECTIONS

**FIGURE 2:**
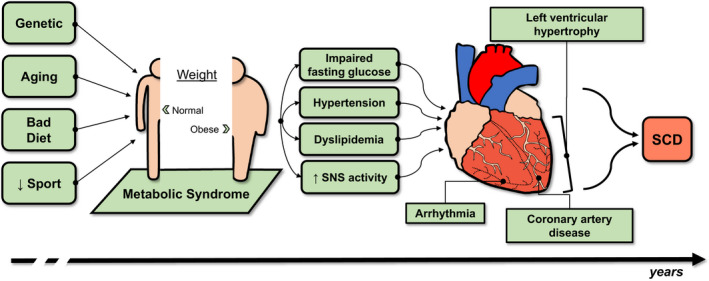
The detrimental effects of metabolic syndrome on the heart. Genetic determinants, but also ageing, diet and reduced physical activity concur in the development of MetS. Such individuals might appear lean even when ‘metabolically obese’. Singularly and taken together, IFG, HTN, DysL and increased sympathetic activity are key elements in MetS pathophysiology with damaging effects on the heart favouring the development of CHD, LVH and AR, eventually causing SCD. However, such associations remain under‐investigated, and the main underlying mechanisms are still poorly understood. SCD, sudden cardiac death; SNS, sympathetic nervous system

MetS is composed of different CV risk factors that mingle together, reinforce each other and cause detrimental effects on several organs, including the heart (Figure 2). As many patients are unaware of having MetS,^172^ also many SCD victims were unaware of having CVDs when SCD occurred.^173^ Even though SCD may happen even in apparently healthy subjects without evident cardiac abnormalities,^174^, ^175^ specific cardiac alterations are often found during post‐mortem investigations.^176^ CAD is considered as the main cause of SCD in subjects over 35 with MetS being one of its major risk factors.^52^ Accordingly, all MetS components are reported to concur to the atherosclerotic process. Of interest, some of them show high rates of association and may need combined treatment strategies as recently discussed for diabetes and obesity.^177^

**FIGURE 3:**
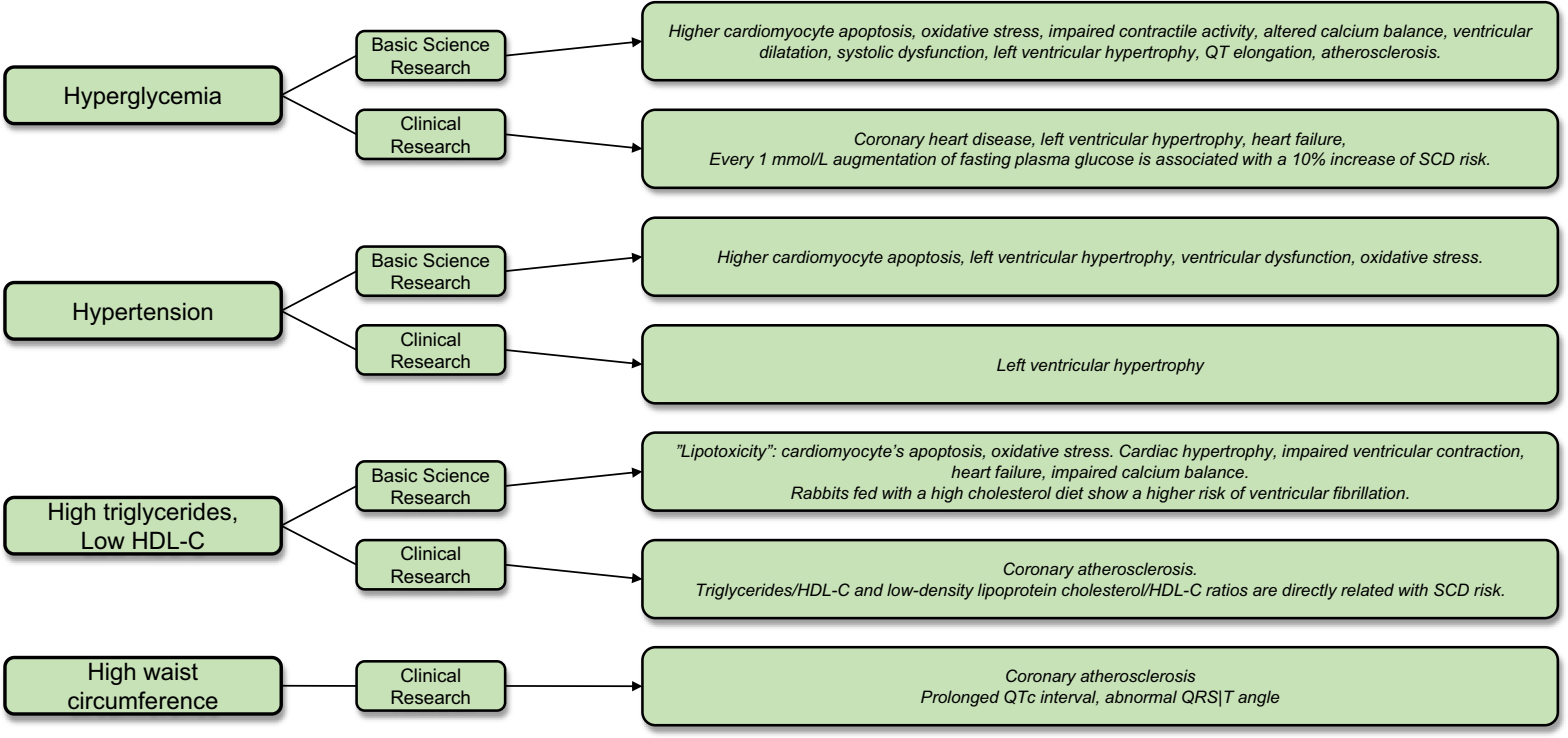
Take‐home figure. HDL‐C, high‐density lipoprotein cholesterol; SCD, sudden cardiac death

Recent studies evaluated the risk of SCD in individuals with MetS and no previous history of CHD suggesting an increased risk of SCD (Table 1).^12^, ^13^, ^14^ Nonetheless, those studies were limited, heterogeneous and far from providing firm conclusions. Specifically, HTN or prescription of anti‐HTN drugs were more frequently reported in victims of SCD in two studies^12^, ^13^ but not in another one.^14^ As stated in the study by Kurl et.al.,^13^ HTN and insulin resistance are probably the key elements to be investigated in order to understand how and why MetS subjects are more likely to be victim of SCD. Meanwhile, WC and low HDL‐C levels might be regarded as predictors for the development of CHD thus predisposing to SCD when underdiagnosed. Furthermore, patients can be lean but be ‘metabolically obese’.^14^ To overcome the limitations of WC, Empana et al.^14^ suggested utilizing sagittal abdominal diameter and they found a higher prevalence of elevated abdominal adiposity in SCD patients. However, few data are available now and further studies are awaited to clarify the correlation between WC and SCD (Figure 3).

**TABLE 1:** Comparison of three studies about sudden cardiac death in patients with metabolic syndrome

	Empana et al.^14^	Kurl et al.^13^	Hess et al.^12^
Type of study	Prospective cohort study	Prospective cohort study	Retrospective cohort study
Patients (n°)	6678	1466	13,168
Populations	European (France)	European (eastern Finland)	US (24.8% blacks)
Male prevalence	100%	100%	44%
Age range	Middle aged	Middle aged	Middle aged
Baseline CHD	no	no	no
Baseline diabetes	no	no	possible
Years of follow‐up	21 (average)	21 (average)	23.6 (median)
MetS criteria	NCEP‐ATP III and IDF	WHO, NCEP‐ATP III, IDF and consensus definition	Consensus definition
MetS prevalence	Up to 16.7%^a^	Up to 38% (men died suddenly out of the hospital)^b^	33.7%
Increased risk for SCD in MetS patients (up to)	68%	160%	70%
Other information	HTN and elevated TGs were similarly reported in both sudden death and control groups. Conversely, larger abdomen adiposity and IFG were more prevalent in sudden death patients.	Men died suddenly out of the hospital or that died suddenly during the follow up had higher systolic and diastolic BP, a higher WC, but lower TGs compared with patients that survived during the follow‐up	HTN or drug treatments for HTN, IFG or drug treatments with insulin or hypoglycaemic agents, and low HDL‐C were independently related to SCD. The number of MetS components was proportionally associated with SCD risk.
Limitations and exceptions	No female and elderly enrolled; No HDL data; SAD substitutes WC	No female and elderly enrolled	No available data for non‐sudden cardiac deaths

Abbreviations: BP, blood pressure; CHD, coronary heart disease; HDL‐C, high‐density lipoprotein cholesterol; HTN, hypertension; IFG, impaired fasting glucose; MetS, metabolic syndrome; SAD, sagittal abdominal diameter; SCD, sudden cardiac death; TG, triglyceride; WC, waist circumference.

^a^ The prevalence of MetS was different depending on different MetS criteria: 14.4% and 16.7% for NCEP‐ATP III and IDF, respectively.

^b^ Using the consensus definition (PMID:IDF‐AHA/NHLBI Joint Interim Statement) of MetS in patients that died suddenly out of hospital. Note that the authors subdivided the patients into three groups: men died suddenly out of the hospital, died suddenly during the follow‐up and survived during the follow‐up.

Concerning other aetiologies, LVH may be of some interest as we reported in the HTN section of this review. MetS subjects have higher incidence of LVH even if not affected by HTN.^178^ Interestingly, a recent study evaluating almost 900 middle‐aged men with 20‐year follow‐up showed that indexed left ventricular mass by body surface area was independently associated with a higher risk of SCD.^179^ On the other hand, evidence on the association between HTN, LVH and SCD is often inconsistent requiring further investigation.^139^, ^140^

The sympathetic nervous system seems to play an important role in the pathogenesis of MetS.^42^, ^43^, ^44^ Worthy of interest, one of the possible triggers for ventricular arrhythmias is the excess of sympathetic hyperstimulation, particularly if cardiac ischaemia is present.^57^ Accordingly, some authors suggested that a higher parasympathetic tone could prevent SCD.^180^, ^181^, ^182^ Thus, physically trained people might have some benefit in terms of SCD prevention, since physical activity could augment the cardiac parasympathetic nerve tone, particularly in the recovery period.^183^ On the other hand, physical activity is related to an augmentation of sympathetic tone during the exercise,^184^ and cases of cardiac arrest during excessive exercise are reported, especially in non‐trained subjects.^185^ In addition, daily low‐intermediate physical activity could play a role in both the prevention and treatment of MetS.^186^, ^187^ Hence, the benefits of a higher parasympathetic tone and physical activity as prevention of both MetS and SCD may be of interest for future analyses.

## 8 CONCLUSIONS

MetS is a common condition predisposing for both higher CV and metabolic risks. Of note, recent evidence shows a possible relationship between MetS and SCD. MetS characteristics such as HG, HTN, high TGs, low HDL‐C, pro‐inflammatory state and excess of sympathetic activity are concurring causes in heart damaging, paving the way towards cardiac alterations that could favour SCD. MetS subjects may have up to 70% of increased risk of SCD. Yet, such an association remains under‐investigated, and the main underlying mechanisms are still poorly understood. The resulting lack of knowledge reflects in the absence of specific targets to reduce SCD risk in this population.

## CONFLICTS OF INTEREST

LL is coinventor on the International Patent (WO/2020/226993) filed in April 2020 and relating to the use of antibodies which specifically bind IL‐1α to reduce various sequelae of ischaemia‐reperfusion injury to the central nervous system. The other authors report no conflict of interest.

## References

[1] Kassi E , Pervanidou P , Kaltsas G , Chrousos G . Metabolic syndrome: definitions and controversies. BMC Med. 2011;9:48. 21542944 10.1186/1741-7015-9-48 PMC3115896

[2] Scuteri A , Laurent S , Cucca F , et al. Metabolic syndrome across Europe: different clusters of risk factors. Eur J Prev Cardiol. 2015;22:486‐491. 24647805 10.1177/2047487314525529 PMC4544872

[3] Pucci G , Alcidi R , Tap L , Battista F , Mattace‐Raso F , Schillaci G . Sex‐ and gender‐related prevalence, cardiovascular risk and therapeutic approach in metabolic syndrome: a review of the literature. Pharmacol Res. 2017;120:34‐42. 28300617 10.1016/j.phrs.2017.03.008

[4] Mottillo S , Filion KB , Genest J , et al. The metabolic syndrome and cardiovascular risk a systematic review and meta‐analysis. J Am Coll Cardiol. 2010;56:1113‐1132. 20863953 10.1016/j.jacc.2010.05.034

[5] Priori SG , Blomström‐Lundqvist C , Mazzanti A , et al. 2015 ESC Guidelines for the management of patients with ventricular arrhythmias and the prevention of sudden cardiac death: The Task Force for the Management of Patients with Ventricular Arrhythmias and the Prevention of Sudden Cardiac Death of the European Society of Cardiology (ESC). Endorsed by: Association for European Paediatric and Congenital Cardiology (AEPC). Eur Heart J. 2015;36:2793‐2867. 26320108 10.1093/eurheartj/ehv316

[6] Deo R , Albert CM . Epidemiology and genetics of sudden cardiac death. Circulation. 2012;125:620‐637. 22294707 10.1161/CIRCULATIONAHA.111.023838 PMC3399522

[7] Mehra R . Global public health problem of sudden cardiac death. J Electrocardiol. 2007;40:S118‐S122. 17993308 10.1016/j.jelectrocard.2007.06.023

[8] Zheng ZJ , Croft JB , Giles WH , Mensah GA . Sudden cardiac death in the United States, 1989 to 1998. Circulation. 2001;104:2158‐2163. 11684624 10.1161/hc4301.098254

[9] Stecker EC , Reinier K , Marijon E , et al. Public health burden of sudden cardiac death in the United States. Circ Arrhythm Electrophysiol. 2014;7:212‐217. 24610738 10.1161/CIRCEP.113.001034 PMC4041478

[10] Galassi A , Reynolds K , He J . Metabolic syndrome and risk of cardiovascular disease: a meta‐analysis. Am J Med. 2006;119:812‐819. 17000207 10.1016/j.amjmed.2006.02.031

[11] Gami AS , Witt BJ , Howard DE , et al. Metabolic syndrome and risk of incident cardiovascular events and death: a systematic review and meta‐analysis of longitudinal studies. J Am Coll Cardiol. 2007;49:403‐414. 17258085 10.1016/j.jacc.2006.09.032

[12] Hess PL , Al‐Khalidi HR , Friedman DJ , et al. The metabolic syndrome and risk of sudden cardiac death: the atherosclerosis risk in communities study. J Am Heart Assoc. 2017;6:e006103. 28835363 10.1161/JAHA.117.006103 PMC5586451

[13] Kurl S , Laaksonen DE , Jae SY , et al. Metabolic syndrome and the risk of sudden cardiac death in middle‐aged men. Int J Cardiol. 2016;203:792‐797. 26595786 10.1016/j.ijcard.2015.10.218

[14] Empana JP , Duciemetiere P , Balkau B , Jouven X . Contribution of the metabolic syndrome to sudden death risk in asymptomatic men: the Paris Prospective Study I. Eur Heart J. 2007;28:1149‐1154. 17428821 10.1093/eurheartj/ehm063

[15] Alberti KG , Eckel RH , Grundy SM , et al. Harmonizing the metabolic syndrome: a joint interim statement of the International Diabetes Federation Task Force on Epidemiology and Prevention; National Heart, Lung, and Blood Institute; American Heart Association; World Heart Federation; International Atherosclerosis Society; and International Association for the Study of Obesity. Circulation. 2009;120:1640‐1645. 19805654 10.1161/CIRCULATIONAHA.109.192644

[16] Dominguez LJ , Barbagallo M . The biology of the metabolic syndrome and aging. Curr Opin Clin Nutr Metab Care. 2016;19:5‐11. 26560521 10.1097/MCO.0000000000000243

[17] Bonomini F , Rodella LF , Rezzani R . Metabolic syndrome, aging and involvement of oxidative stress. Aging Dis. 2015;6:109‐120. 25821639 10.14336/AD.2014.0305 PMC4365955

[18] Stančáková A , Laakso M . Genetics of metabolic syndrome. Rev Endocr Metab Disord. 2014;15:243‐252. 25124343 10.1007/s11154-014-9293-9

[19] Abou Ziki MD , Mani A . Metabolic syndrome: genetic insights into disease pathogenesis. Curr Opin Lipidol. 2016;27:162‐171. 26825138 10.1097/MOL.0000000000000276 PMC5141383

[20] Ouchi N , Parker JL , Lugus JJ , Walsh K . Adipokines in inflammation and metabolic disease. Nat Rev Immunol. 2011;11:85‐97. 21252989 10.1038/nri2921 PMC3518031

[21] Liberale L , Montecucco F . Adipocytokines and cardiovascular diseases: putative role of neuregulin 4. Eur J Clin Invest. 2020;50(8):e13306. 10.1111/eci.13306 32511758

[22] Liberale L , Bertolotto M , Carbone F , et al. Resistin exerts a beneficial role in atherosclerotic plaque inflammation by inhibiting neutrophil migration. Int J Cardiol. 2018;272:13‐19. 30075966 10.1016/j.ijcard.2018.07.112

[23] Liberale L , Carbone F , Bertolotto M , et al. Serum adiponectin levels predict acute coronary syndrome (ACS) in patients with severe carotid stenosis. Vascul Pharmacol. 2018;102:37‐43. 29305337 10.1016/j.vph.2017.12.066

[24] Liberale L , Bonaventura A , Vecchiè A , et al. The role of adipocytokines in coronary atherosclerosis. Curr Atheroscler Rep. 2017;19:10. 28185154 10.1007/s11883-017-0644-3

[25] Frühbeck G , Catalán V , Rodríguez A , et al. Involvement of the leptin‐adiponectin axis in inflammation and oxidative stress in the metabolic syndrome. Sci Rep. 2017;7:6619. 28747790 10.1038/s41598-017-06997-0 PMC5529549

[26] Liberale L , Montecucco F , Tardif JC , Libby P , Camici GG . Inflamm‐ageing: the role of inflammation in age‐dependent cardiovascular disease. Eur Heart J. 2020;41:2974‐2982. 32006431 10.1093/eurheartj/ehz961 PMC7453832

[27] Franceschi C , Garagnani P , Parini P , Giuliani C , Santoro A . Inflammaging: a new immune‐metabolic viewpoint for age‐related diseases. Nat Rev Endocrinol. 2018;14:576‐590. 30046148 10.1038/s41574-018-0059-4

[28] Després J‐P , Lemieux I . Abdominal obesity and metabolic syndrome. Nature. 2006;444:881‐887. 17167477 10.1038/nature05488

[29] Grundy SM . Adipose tissue and metabolic syndrome: too much, too little or neither. Eur J Clin Invest. 2015;45:1209‐1217. 26291691 10.1111/eci.12519 PMC5049481

[30] Vecchié A , Dallegri F , Carbone F , et al. Obesity phenotypes and their paradoxical association with cardiovascular diseases. Eur J Intern Med. 2018;48:6‐17. 29100895 10.1016/j.ejim.2017.10.020

[31] Elffers TW , de Mutsert R , Lamb HJ , et al. Body fat distribution, in particular visceral fat, is associated with cardiometabolic risk factors in obese women. PLoS One. 2017;12(9):e0185403. 28957363 10.1371/journal.pone.0185403 PMC5619737

[32] Pak K , Lee SH , Lee JG , Seok JW , Kim IJ . Comparison of visceral fat measures with cardiometabolic risk factors in healthy adults. PLoS One. 2016;11:e0153031. 27043708 10.1371/journal.pone.0153031 PMC4820273

[33] Wajchenberg BL . Subcutaneous and visceral adipose tissue: their relation to the metabolic syndrome. Endocr Rev. 2000;21:697‐738. 11133069 10.1210/edrv.21.6.0415

[34] Ibrahim MM . Subcutaneous and visceral adipose tissue: structural and functional differences. Obes Rev. 2010;11:11‐18. 19656312 10.1111/j.1467-789X.2009.00623.x

[35] Kwon H , Kim D , Kim JS . Body fat distribution and the risk of incident metabolic syndrome: a longitudinal cohort study. Sci Rep. 2017;7:10955. 28887474 10.1038/s41598-017-09723-y PMC5591218

[36] Lee S , Kuk JL , Kim Y , Arslanian SA . Measurement site of visceral adipose tissue and prediction of metabolic syndrome in youth. Pediatr Diabetes. 2011;12:250‐257. 21129140 10.1111/j.1399-5448.2010.00705.x PMC3426866

[37] Demerath EW , Reed D , Rogers N , et al. Visceral adiposity and its anatomical distribution as predictors of the metabolic syndrome and cardiometabolic risk factor levels. Am J Clin Nutr. 2008;88:1263‐1271. 18996861 10.3945/ajcn.2008.26546 PMC2801427

[38] Kuk JL , Church TS , Blair SN , Ross R . Does measurement site for visceral and abdominal subcutaneous adipose tissue alter associations with the metabolic syndrome? Diabetes Care. 2006;29:679‐684. 16505526 10.2337/diacare.29.03.06.dc05-1500

[39] Rochlani Y , Pothineni NV , Kovelamudi S , Mehta JL . Metabolic syndrome: pathophysiology, management, and modulation by natural compounds. Ther Adv Cardiovasc Dis. 2017;11:215‐225. 28639538 10.1177/1753944717711379 PMC5933580

[40] Feldman RD , Bierbrier GS . Insulin‐mediated vasodilation: impairment with increased blood pressure and body mass. Lancet. 1993;342:707‐709. 8103822 10.1016/0140-6736(93)91708-t

[41] Sarzani R , Salvi F , Dessì‐Fulgheri P , Rappelli A . Renin‐angiotensin system, natriuretic peptides, obesity, metabolic syndrome, and hypertension: an integrated view in humans. J Hypertens. 2008;26:831‐843. 18398321 10.1097/HJH.0b013e3282f624a0

[42] Thorp AA , Schlaich MP . Relevance of sympathetic nervous system activation in obesity and metabolic syndrome. J Diabetes Res. 2015;2015:341583. 26064978 10.1155/2015/341583 PMC4430650

[43] Grassi G , Quarti‐Trevano F , Seravalle G , Dell'Oro R . Cardiovascular risk and adrenergic overdrive in the metabolic syndrome. Nutr Metab Cardiovasc Dis. 2007;17:473‐481. 17451923 10.1016/j.numecd.2007.01.004

[44] Mancia G , Bousquet P , Elghozi JL , et al. The sympathetic nervous system and the metabolic syndrome. J Hypertens. 2007;25:909‐920. 17414649 10.1097/HJH.0b013e328048d004

[45] Saxena PR . Interaction between the renin‐angiotensin‐aldosterone and sympathetic nervous systems. J Cardiovasc Pharmacol. 1992;19(Suppl 6):S80‐S88. 1382170 10.1097/00005344-199219006-00013

[46] Jouven X , Lemaître RN , Rea TD , Sotoodehnia N , Empana J‐P , Siscovick DS . Diabetes, glucose level, and risk of sudden cardiac death. Eur Heart J. 2005;26:2142‐2147. 15980034 10.1093/eurheartj/ehi376

[47] Messerli FH . Hypertension and sudden cardiac death. Am J Hypertens. 1999;12:181S‐S188. 10619570 10.1016/s0895-7061(99)00106-5

[48] Shenasa M , Shenasa H . Hypertension, left ventricular hypertrophy, and sudden cardiac death. Int J Cardiol. 2017;237:60‐63. 28285801 10.1016/j.ijcard.2017.03.002

[49] Myerburg RJ , Junttila MJ . Sudden cardiac death caused by coronary heart disease. Circulation. 2012;125:1043‐1052. 22371442 10.1161/CIRCULATIONAHA.111.023846

[50] Chugh SS , Kelly KL , Titus JL . Sudden cardiac death with apparently normal heart. Circulation. 2000;102:649‐654. 10931805 10.1161/01.cir.102.6.649

[51] Katritsis DG , Gersh BJ , Camm AJ . A clinical perspective on sudden cardiac death. Arrhythm Electrophysiol Rev. 2016;5:177‐182. 28116082 10.15420/aer.2016:11:2 PMC5248660

[52] Hayashi M , Shimizu W , Albert CM . The spectrum of epidemiology underlying sudden cardiac death. Circ Res. 2015;116:1887‐1906. 26044246 10.1161/CIRCRESAHA.116.304521 PMC4929621

[53] Montagnana M , Lippi G , Franchini M , Targher G , Cesare Guidi G . Sudden cardiac death: prevalence, pathogenesis, and prevention. Ann Med. 2008;40:360‐375. 18484348 10.1080/07853890801964930

[54] Goyal V , Jassal DS , Dhalla NS . Pathophysiology and prevention of sudden cardiac death. Can J Physiol Pharmacol. 2016;94:237‐244. 26651385 10.1139/cjpp-2015-0366

[55] Franciosi S , Perry FKG , Roston TM , Armstrong KR , Claydon VE , Sanatani S . The role of the autonomic nervous system in arrhythmias and sudden cardiac death. Auton Neurosci. 2017;205:1‐11. 28392310 10.1016/j.autneu.2017.03.005

[56] Schwartz PJ , Ackerman MJ , Antzelevitch C , et al. Inherited cardiac arrhythmias. Nat Rev Dis Primers. 2020;6:58. 32678103 10.1038/s41572-020-0188-7 PMC7935690

[57] Shen MJ , Zipes DP . Role of the autonomic nervous system in modulating cardiac arrhythmias. Circ Res. 2014;114:1004‐1021. 24625726 10.1161/CIRCRESAHA.113.302549

[58] Chistiakov DA , Ashwell KW , Orekhov AN , Bobryshev YV . Innervation of the arterial wall and its modification in atherosclerosis. Auton Neurosci. 2015;193:7‐11. 26164815 10.1016/j.autneu.2015.06.005

[59] Remme WJ . The sympathetic nervous system and ischaemic heart disease. Eur Heart J. 1998;(19 Suppl F):F62‐F71. 9651738

[60] American Diabetes A . 2. Classification and diagnosis of diabetes: standards of medical care in diabetes‐2020. Diabetes Care. 2020;43:S14‐S31. 31862745 10.2337/dc20-S002

[61] Bansal N . Prediabetes diagnosis and treatment: a review. World J Diabetes. 2015;6:296‐303. 25789110 10.4239/wjd.v6.i2.296 PMC4360422

[62] Annapurna A , Reddy CS , Akondi RB , Rao SR . Cardioprotective actions of two bioflavonoids, quercetin and rutin, in experimental myocardial infarction in both normal and streptozotocin‐induced type I diabetic rats. J Pharm Pharmacol. 2009;61:1365‐1374. 19814870 10.1211/jpp/61.10.0014

[63] Mapanga RF , Joseph D , Symington B , et al. Detrimental effects of acute hyperglycaemia on the rat heart. Acta Physiol (Oxf). 2014;210:546‐564. 24286628 10.1111/apha.12184

[64] Qiu Z , Lei S , Zhao B , et al. NLRP3 inflammasome activation‐mediated pyroptosis aggravates myocardial ischemia/reperfusion injury in diabetic rats. Oxid Med Cell Longev. 2017;2017:9743280. 29062465 10.1155/2017/9743280 PMC5618779

[65] Zhang B , Zhai M , Li B , et al. Honokiol ameliorates myocardial ischemia/reperfusion injury in type 1 diabetic rats by reducing oxidative stress and apoptosis through activating the SIRT1‐Nrf2 signaling pathway. Oxid Med Cell Longev. 2018;2018:3159801. 29675132 10.1155/2018/3159801 PMC5838504

[66] Cai L , Li W , Wang G , Guo L , Jiang Y , Kang YJ . Hyperglycemia‐induced apoptosis in mouse myocardium: mitochondrial cytochrome C‐mediated caspase‐3 activation pathway. Diabetes. 2002;51:1938‐1948. 12031984 10.2337/diabetes.51.6.1938

[67] Yu T , Sheu SS , Robotham JL , Yoon Y . Mitochondrial fission mediates high glucose‐induced cell death through elevated production of reactive oxygen species. Cardiovasc Res. 2008;79:341‐351. 18440987 10.1093/cvr/cvn104 PMC2646899

[68] Li Y , Li Y , Feng Q , Arnold M , Peng T . Calpain activation contributes to hyperglycaemia‐induced apoptosis in cardiomyocytes. Cardiovasc Res. 2009;84:100‐110. 19505932 10.1093/cvr/cvp189

[69] Zhang X , Lin Q , Chen J , et al. High glucose‐induced cardiomyocyte death may be linked to unbalanced branched‐chain amino acids and energy metabolism. Molecules. 2018;23(4):807. 10.3390/molecules23040807 PMC6017930 29614759

[70] Sorrentino A , Borghetti G , Zhou Y , et al. Hyperglycemia induces defective Ca2+ homeostasis in cardiomyocytes. Am J Physiol Heart Circ Physiol. 2017;312:H150‐H161. 27881388 10.1152/ajpheart.00737.2016 PMC5283918

[71] Ren J , Gintant GA , Miller RE , Davidoff AJ . High extracellular glucose impairs cardiac E‐C coupling in a glycosylation‐dependent manner. Am J Physiol. 1997;273:H2876‐H2883. 9435627 10.1152/ajpheart.1997.273.6.H2876

[72] Nunes S , Soares E , Fernandes J , et al. Early cardiac changes in a rat model of prediabetes: brain natriuretic peptide overexpression seems to be the best marker. Cardiovasc Diabetol. 2013;12:44. 23497124 10.1186/1475-2840-12-44 PMC3599663

[73] D'Amico M , Marfella R , Nappo F , et al. High glucose induces ventricular instability and increases vasomotor tone in rats. Diabetologia. 2001;44:464‐470. 11357477 10.1007/s001250051644

[74] Aronson D , Rayfield EJ . How hyperglycemia promotes atherosclerosis: molecular mechanisms. Cardiovasc Diabetol. 2002;1:1. 12119059 10.1186/1475-2840-1-1 PMC116615

[75] Bornfeldt KE , Tabas I . Insulin resistance, hyperglycemia, and atherosclerosis. Cell Metab. 2011;14:575‐585. 22055501 10.1016/j.cmet.2011.07.015 PMC3217209

[76] Hegab Z , Gibbons S , Neyses L , Mamas MA . Role of advanced glycation end products in cardiovascular disease. World J Cardiol. 2012;4:90‐102. 22558488 10.4330/wjc.v4.i4.90 PMC3342583

[77] Fishman SL , Sonmez H , Basman C , Singh V , Poretsky L . The role of advanced glycation end‐products in the development of coronary artery disease in patients with and without diabetes mellitus: a review. Mol Med. 2018;24:59. 30470170 10.1186/s10020-018-0060-3 PMC6251169

[78] Yang P , Feng J , Peng Q , Liu X , Fan Z . Advanced glycation end products: potential mechanism and therapeutic target in cardiovascular complications under diabetes. Oxid Med Cell Longev. 2019;2019:9570616. 31885827 10.1155/2019/9570616 PMC6925928

[79] Huang Y , Cai X , Chen P , et al. Associations of prediabetes with all‐cause and cardiovascular mortality: a meta‐analysis. Ann Med. 2014;46:684‐692. 25230915 10.3109/07853890.2014.955051

[80] Aune D , Schlesinger S , Norat T , Riboli E . Diabetes mellitus and the risk of sudden cardiac death: a systematic review and meta‐analysis of prospective studies. Nutr Metab Cardiovasc Dis. 2018;28:543‐556. 29730085 10.1016/j.numecd.2018.02.011

[81] Laukkanen JA , Makikallio TH , Ronkainen K , Karppi J , Kurl S . Impaired fasting plasma glucose and type 2 diabetes are related to the risk of out‐of‐hospital sudden cardiac death and all‐cause mortality. Diabetes Care. 2013;36:1166‐1171. 23248190 10.2337/dc12-0110 PMC3631879

[82] Patel RB , Moorthy MV , Chiuve SE , Pradhan AD , Cook NR , Albert CM . Hemoglobin A1c levels and risk of sudden cardiac death: a nested case‐control study. Heart Rhythm. 2017;14:72‐78. 27591826 10.1016/j.hrthm.2016.08.044 PMC5754013

[83] Waks JW , Haq KT , Tompkins C , et al. Competing risks in patients with primary prevention implantable cardioverter‐defibrillators: Global Electrical Heterogeneity and Clinical Outcomes study. Heart Rhythm. 2021;18:977‐986. 33684549 10.1016/j.hrthm.2021.03.006 PMC8169548

[84] Siscovick DS , Sotoodehnia N , Rea TD , Raghunathan TE , Jouven X , Lemaitre RN . Type 2 diabetes mellitus and the risk of sudden cardiac arrest in the community. Rev Endocr Metab Disord. 2010;11:53‐59. 20195771 10.1007/s11154-010-9133-5 PMC3413310

[85] van Noord C , Sturkenboom MC , Straus SM , et al. Serum glucose and insulin are associated with QTc and RR intervals in nondiabetic elderly. Eur J Endocrinol. 2010;162:241‐248. 19897609 10.1530/EJE-09-0878

[86] Laakso M . Glycemic control and the risk for coronary heart disease in patients with non‐insulin‐dependent diabetes mellitus. The Finnish Studies. Ann Intern Med. 1996;124:127‐130. 8554204 10.7326/0003-4819-124-1_part_2-199601011-00009

[87] Su G , Mi S , Tao H , et al. Association of glycemic variability and the presence and severity of coronary artery disease in patients with type 2 diabetes. Cardiovasc Diabetol. 2011;10:19. 21349201 10.1186/1475-2840-10-19 PMC3056765

[88] Merino J , Leong A , Posner DC , et al. Genetically driven hyperglycemia increases risk of coronary artery disease separately from type 2 diabetes. Diabetes Care. 2017;40:687‐693. 28298470 10.2337/dc16-2625 PMC5399655

[89] Cho YR , Ann SH , Won KB , et al. Association between insulin resistance, hyperglycemia, and coronary artery disease according to the presence of diabetes. Sci Rep. 2019;9:6129. 31477741 10.1038/s41598-019-42700-1 PMC6718672

[90] Kitazawa M , Fujihara K , Osawa T , et al. Risk of coronary artery disease according to glucose abnormality status and prior coronary artery disease in Japanese men. Metabolism. 2019;101:153991. 31666194 10.1016/j.metabol.2019.153991

[91] Mente A , de Koning L , Shannon HS , Anand SS . A systematic review of the evidence supporting a causal link between dietary factors and coronary heart disease. Arch Intern Med. 2009;169:659‐669. 19364995 10.1001/archinternmed.2009.38

[92] Fan J , Song Y , Wang Y , Hui R , Zhang W . Dietary glycemic index, glycemic load, and risk of coronary heart disease, stroke, and stroke mortality: a systematic review with meta‐analysis. PLoS One. 2012;7:e52182. 23284926 10.1371/journal.pone.0052182 PMC3527433

[93] Lin TH , Chiu HC , Su HM , et al. Association between fasting plasma glucose and left ventricular mass and left ventricular hypertrophy over 4 years in a healthy population aged 60 and older. J Am Geriatr Soc. 2007;55:717‐724. 17493191 10.1111/j.1532-5415.2007.01134.x

[94] Diederichsen SZ , Pareek M , Nielsen ML , et al. Impact of fasting glucose on electrocardiographic left ventricular hypertrophy in an elderly general population. Blood Press. 2015;24:164‐173. 25881496 10.3109/08037051.2015.1030892

[95] Cai X , Liu X , Sun L , et al. Prediabetes and the risk of heart failure: a meta‐analysis. Diabetes Obes Metab. 2021;23(8):1746‐1753. 33769672 10.1111/dom.14388

[96] Nielson C , Lange T . Blood glucose and heart failure in nondiabetic patients. Diabetes Care. 2005;28:607‐611. 15735196 10.2337/diacare.28.3.607

[97] From AM , Scott CG , Chen HH . The development of heart failure in patients with diabetes mellitus and pre‐clinical diastolic dysfunction a population‐based study. J Am Coll Cardiol. 2010;55:300‐305. 20117433 10.1016/j.jacc.2009.12.003 PMC3878075

[98] McMurray JJ , Gerstein HC , Holman RR , Pfeffer MA . Heart failure: a cardiovascular outcome in diabetes that can no longer be ignored. Lancet Diabetes Endocrinol. 2014;2:843‐851. 24731668 10.1016/S2213-8587(14)70031-2

[99] Bouthoorn S , Valstar GB , Gohar A , et al. The prevalence of left ventricular diastolic dysfunction and heart failure with preserved ejection fraction in men and women with type 2 diabetes: a systematic review and meta‐analysis. Diab Vasc Dis Res. 2018;15:477‐493. 30037278 10.1177/1479164118787415 PMC6236645

[100] Sliem H , Nasr G . Left ventricular structure and function in prediabetic adults: relationship with insulin resistance. J Cardiovasc Dis Res. 2011;2:23‐28. 21716748 10.4103/0975-3583.78583 PMC3120268

[101] Bamaiyi AJ , Woodiwiss AJ , Peterson V , et al. Insulin resistance influences the impact of hypertension on left ventricular diastolic dysfunction in a community sample. Clin Cardiol. 2019;42:305‐311. 30592058 10.1002/clc.23145 PMC6712340

[102] Jia G , DeMarco VG , Sowers JR . Insulin resistance and hyperinsulinaemia in diabetic cardiomyopathy. Nat Rev Endocrinol. 2016;12:144‐153. 26678809 10.1038/nrendo.2015.216 PMC4753054

[103] Cittadini A , Napoli R , Monti MG , et al. Metformin prevents the development of chronic heart failure in the SHHF rat model. Diabetes. 2012;61:944‐953. 22344560 10.2337/db11-1132 PMC3314362

[104] Williams B , Mancia G , Spiering W , et al. 2018 ESC/ESH Guidelines for the management of arterial hypertension. Eur Heart J. 2018;39:3021‐3104. 30165516 10.1093/eurheartj/ehy339

[105] Tocci G , Nati G , Cricelli C , et al. Prevalence and control of hypertension in the general practice in Italy: updated analysis of a large database. J Hum Hypertens. 2017;31:258‐262. 27629243 10.1038/jhh.2016.71

[106] Fortuno MA , Ravassa S , Fortuno A , Zalba G , Diez J . Cardiomyocyte apoptotic cell death in arterial hypertension: mechanisms and potential management. Hypertension. 2001;38:1406‐1412. 11751726 10.1161/hy1201.099615

[107] Fu YC , Chi CS , Yin SC , Hwang B , Chiu YT , Hsu SL . Norepinephrine induces apoptosis in neonatal rat cardiomyocytes through a reactive oxygen species‐TNF alpha‐caspase signaling pathway. Cardiovasc Res. 2004;62:558‐567. 15158148 10.1016/j.cardiores.2004.01.039

[108] Li Z , Bing OH , Long X , Robinson KG , Lakatta EG . Increased cardiomyocyte apoptosis during the transition to heart failure in the spontaneously hypertensive rat. Am J Physiol. 1997;272:H2313‐H2319. 9176300 10.1152/ajpheart.1997.272.5.H2313

[109] Condorelli G , Morisco C , Stassi G , et al. Increased cardiomyocyte apoptosis and changes in proapoptotic and antiapoptotic genes bax and bcl‐2 during left ventricular adaptations to chronic pressure overload in the rat. Circulation. 1999;99:3071‐3078. 10368127 10.1161/01.cir.99.23.3071

[110] Dolinsky VW , Chakrabarti S , Pereira TJ , et al. Resveratrol prevents hypertension and cardiac hypertrophy in hypertensive rats and mice. Biochim Biophys Acta. 2013;1832:1723‐1733. 23707558 10.1016/j.bbadis.2013.05.018

[111] Hernandez AM , Huber JS , Murphy ST , et al. Longitudinal evaluation of left ventricular substrate metabolism, perfusion, and dysfunction in the spontaneously hypertensive rat model of hypertrophy using small‐animal PET/CT imaging. J Nucl Med. 2013;54:1938‐1945. 24092939 10.2967/jnumed.113.120105 PMC4000452

[112] Tang Y , Mi C , Liu J , Gao F , Long J . Compromised mitochondrial remodeling in compensatory hypertrophied myocardium of spontaneously hypertensive rat. Cardiovasc Pathol. 2014;23:101‐106. 24388463 10.1016/j.carpath.2013.11.002

[113] Kwiecinski J , Lennen RJ , Gray GA , et al. Progression and regression of left ventricular hypertrophy and myocardial fibrosis in a mouse model of hypertension and concomitant cardiomyopathy. J Cardiovasc Magn Reson. 2020;22:57. 32758255 10.1186/s12968-020-00655-7 PMC7409657

[114] Li J , Kemp BA , Howell NL , et al. Metabolic changes in spontaneously hypertensive rat hearts precede cardiac dysfunction and left ventricular hypertrophy. J Am Heart Assoc. 2019;8:e010926. 30764689 10.1161/JAHA.118.010926 PMC6405673

[115] Oliver PM , Fox JE , Kim R , et al. Hypertension, cardiac hypertrophy, and sudden death in mice lacking natriuretic peptide receptor A. Proc Natl Acad Sci USA. 1997;94:14730‐14735. 9405681 10.1073/pnas.94.26.14730 PMC25105

[116] Haider AW , Larson MG , Benjamin EJ , Levy D . Increased left ventricular mass and hypertrophy are associated with increased risk for sudden death. J Am Coll Cardiol. 1998;32:1454‐1459. 9809962 10.1016/s0735-1097(98)00407-0

[117] Paoletti E , Specchia C , Di Maio G , et al. The worsening of left ventricular hypertrophy is the strongest predictor of sudden cardiac death in haemodialysis patients: a 10 year survey. Nephrol Dial Transplant. 2004;19:1829‐1834. 15128888 10.1093/ndt/gfh288

[118] Kahan T , Bergfeldt L . Left ventricular hypertrophy in hypertension: its arrhythmogenic potential. Heart. 2005;91:250‐256. 15657259 10.1136/hrt.2004.042473 PMC1768675

[119] Xiao HD , Fuchs S , Campbell DJ , et al. Mice with cardiac‐restricted angiotensin‐converting enzyme (ACE) have atrial enlargement, cardiac arrhythmia, and sudden death. Am J Pathol. 2004;165:1019‐1032. 15331425 10.1016/S0002-9440(10)63363-9 PMC1618615

[120] Mazzolai L , Pedrazzini T , Nicoud F , Gabbiani G , Brunner HR , Nussberger J . Increased cardiac angiotensin II levels induce right and left ventricular hypertrophy in normotensive mice. Hypertension. 2000;35:985‐991. 10775573 10.1161/01.hyp.35.4.985

[121] Huggins CE , Domenighetti AA , Pedrazzini T , Pepe S , Delbridge LM . Elevated intracardiac angiotensin II leads to cardiac hypertrophy and mechanical dysfunction in normotensive mice. J Renin Angiotensin Aldosterone Syst. 2003;4:186‐190. 14608525 10.3317/jraas.2003.030

[122] Zhang C , Yasuno S , Kuwahara K , et al. Blockade of angiotensin II type 1 receptor improves the arrhythmia morbidity in mice with left ventricular hypertrophy. Circ J. 2006;70:335‐341. 16501302 10.1253/circj.70.335

[123] Sovari AA , Rutledge CA , Jeong EM , et al. Mitochondria oxidative stress, connexin43 remodeling, and sudden arrhythmic death. Circ Arrhythm Electrophysiol. 2013;6:623‐631. 23559673 10.1161/CIRCEP.112.976787 PMC3716298

[124] Griendling KK , Ushio‐Fukai M . Reactive oxygen species as mediators of angiotensin II signaling. Regul Pept. 2000;91:21‐27. 10967199 10.1016/s0167-0115(00)00136-1

[125] Touyz RM . Reactive oxygen species and angiotensin II signaling in vascular cells – implications in cardiovascular disease. Braz J Med Biol Res. 2004;37:1263‐1273. 15273829 10.1590/s0100-879x2004000800018

[126] Jeong EM , Liu M , Sturdy M , et al. Metabolic stress, reactive oxygen species, and arrhythmia. J Mol Cell Cardiol. 2012;52:454‐463. 21978629 10.1016/j.yjmcc.2011.09.018 PMC3264827

[127] Laukkanen JA , Jennings JR , Kauhanen J , Makikallio TH , Ronkainen K , Kurl S . Relation of systemic blood pressure to sudden cardiac death. Am J Cardiol. 2012;110:378‐382. 22521306 10.1016/j.amjcard.2012.03.035

[128] Rapsomaniki E , Timmis A , George J , et al. Blood pressure and incidence of twelve cardiovascular diseases: lifetime risks, healthy life‐years lost, and age‐specific associations in 1.25 million people. Lancet. 2014;383:1899‐1911. 24881994 10.1016/S0140-6736(14)60685-1 PMC4042017

[129] Katibi IA . Left ventricular hypertrophy and hypertension. Niger J Med. 2004;13:8‐17. 15296101

[130] Reinier K , Nichols GA , Huertas‐Vazquez A , et al. Distinctive clinical profile of blacks versus whites presenting with sudden cardiac arrest. Circulation. 2015;132:380‐387. 26240262 10.1161/CIRCULATIONAHA.115.015673 PMC4526124

[131] Cunningham KS , Spears DA , Care M . Evaluation of cardiac hypertrophy in the setting of sudden cardiac death. Forensic Sci Res. 2019;4:223‐240. 31489388 10.1080/20961790.2019.1633761 PMC6713129

[132] McLenachan JM , Dargie HJ . Ventricular arrhythmias in hypertensive left ventricular hypertrophy. Relationship to coronary artery disease, left ventricular dysfunction, and myocardial fibrosis. Am J Hypertens. 1990;3:735‐740. 2145865 10.1093/ajh/3.10.735

[133] Diez J . Mechanisms of cardiac fibrosis in hypertension. J Clin Hypertens (Greenwich). 2007;9:546‐550. 17617765 10.1111/j.1524-6175.2007.06626.x PMC8110048

[134] de Jong S , van Veen TA , van Rijen HV , de Bakker JM . Fibrosis and cardiac arrhythmias. J Cardiovasc Pharmacol. 2011;57:630‐638. 21150449 10.1097/FJC.0b013e318207a35f

[135] Nguyen TP , Qu Z , Weiss JN . Cardiac fibrosis and arrhythmogenesis: the road to repair is paved with perils. J Mol Cell Cardiol. 2014;70:83‐91. 24184999 10.1016/j.yjmcc.2013.10.018 PMC3995831

[136] Shenasa M . Fibrosis and ventricular arrhythmogenesis: role of cardiac MRI. Card Electrophysiol Clin. 2019;11:551‐562. 31400878 10.1016/j.ccep.2019.06.002

[137] Cuspidi C , Facchetti R , Bombelli M , et al. High normal blood pressure and left ventricular hypertrophy echocardiographic findings from the PAMELA population. Hypertension. 2019;73:612‐619. 30612493 10.1161/HYPERTENSIONAHA.118.12114

[138] Hendriks T , Said MA , Janssen LMA , et al. Effect of systolic blood pressure on left ventricular structure and function: a mendelian randomization study. Hypertension. 2019;74:826‐832. 31476911 10.1161/HYPERTENSIONAHA.119.12679

[139] Dunn FG , Pringle SD . Sudden cardiac death, ventricular arrhythmias and hypertensive left ventricular hypertrophy. J Hypertens. 1993;11:1003‐1010. 8258663 10.1097/00004872-199310000-00002

[140] Pringle SD , Dunn FG , Macfarlane PW , McKillop JH , Lorimer AR , Cobbe SM . Significance of ventricular arrhythmias in systemic hypertension with left ventricular hypertrophy. Am J Cardiol. 1992;69:913‐917. 1532285 10.1016/0002-9149(92)90792-w

[141] Nishimura M , Tokoro T , Nishida M , et al. Sympathetic overactivity and sudden cardiac death among hemodialysis patients with left ventricular hypertrophy. Int J Cardiol. 2010;142:80‐86. 19168236 10.1016/j.ijcard.2008.12.104

[142] Kadowaki T , Sekikawa A , Murata K , et al. Japanese men have larger areas of visceral adipose tissue than Caucasian men in the same levels of waist circumference in a population‐based study. Int J Obes (Lond). 2006;30:1163‐1165. 16446744 10.1038/sj.ijo.0803248

[143] Carroll JF , Chiapa AL , Rodriquez M , et al. Visceral fat, waist circumference, and BMI: impact of race/ethnicity. Obesity (Silver Spring). 2008;16:600‐607. 18239557 10.1038/oby.2007.92

[144] Fisher EA , Feig JE , Hewing B , Hazen SL , Smith JD . High‐density lipoprotein function, dysfunction, and reverse cholesterol transport. Arterioscler Thromb Vasc Biol. 2012;32:2813‐2820. 23152494 10.1161/ATVBAHA.112.300133 PMC3501261

[145] Houten SM , Wanders RJ . A general introduction to the biochemistry of mitochondrial fatty acid beta‐oxidation. J Inherit Metab Dis. 2010;33:469‐477. 20195903 10.1007/s10545-010-9061-2 PMC2950079

[146] Sharrett AR , Ballantyne CM , Coady SA , et al. Coronary heart disease prediction from lipoprotein cholesterol levels, triglycerides, lipoprotein(a), apolipoproteins A‐I and B, and HDL density subfractions: the Atherosclerosis Risk in Communities (ARIC) study. Circulation. 2001;104:1108‐1113. 11535564 10.1161/hc3501.095214

[147] Lopaschuk GD , Ussher JR , Folmes CD , Jaswal JS , Stanley WC . Myocardial fatty acid metabolism in health and disease. Physiol Rev. 2010;90:207‐258. 20086077 10.1152/physrev.00015.2009

[148] Lopaschuk GD , Folmes CD , Stanley WC . Cardiac energy metabolism in obesity. Circ Res. 2007;101:335‐347. 17702980 10.1161/CIRCRESAHA.107.150417

[149] Ge F , Hu C , Hyodo E , et al. Cardiomyocyte triglyceride accumulation and reduced ventricular function in mice with obesity reflect increased long chain Fatty Acid uptake and de novo Fatty Acid synthesis. J Obes. 2012;2012:205648. 22132320 10.1155/2012/205648 PMC3216284

[150] Drosatos K , Schulze PC . Cardiac lipotoxicity: molecular pathways and therapeutic implications. Curr Heart Fail Rep. 2013;10:109‐121. 23508767 10.1007/s11897-013-0133-0 PMC3647019

[151] Costantino S , Akhmedov A , Melina G , et al. Obesity‐induced activation of JunD promotes myocardial lipid accumulation and metabolic cardiomyopathy. Eur Heart J. 2019;40:997‐1008. 30629164 10.1093/eurheartj/ehy903

[152] He L , Kim T , Long Q , et al. Carnitine palmitoyltransferase‐1b deficiency aggravates pressure overload‐induced cardiac hypertrophy caused by lipotoxicity. Circulation. 2012;126:1705‐1716. 22932257 10.1161/CIRCULATIONAHA.111.075978 PMC3484985

[153] Tucci S , Flogel U , Sturm M , Borsch E , Spiekerkoetter U . Disrupted fat distribution and composition due to medium‐chain triglycerides in mice with a beta‐oxidation defect. Am J Clin Nutr. 2011;94:439‐449. 21697078 10.3945/ajcn.111.012948

[154] Sanchez G , Araneda F , Pena JP , et al. High‐fat‐diet‐induced obesity produces spontaneous ventricular arrhythmias and increases the activity of ryanodine receptors in mice. Int J Mol Sci. 2018;19:533. 10.3390/ijms19020533 PMC5855755 29439404

[155] Liu YB , Wu CC , Lu LS , et al. Sympathetic nerve sprouting, electrical remodeling, and increased vulnerability to ventricular fibrillation in hypercholesterolemic rabbits. Circ Res. 2003;92:1145‐1152. 12714561 10.1161/01.RES.0000072999.51484.92

[156] Liu YB , Lee YT , Pak HN , et al. Effects of simvastatin on cardiac neural and electrophysiologic remodeling in rabbits with hypercholesterolemia. Heart Rhythm. 2009;6:69‐75. 19121803 10.1016/j.hrthm.2008.10.004 PMC2757294

[157] Aune D , Schlesinger S , Norat T , Riboli E . Body mass index, abdominal fatness, and the risk of sudden cardiac death: a systematic review and dose‐response meta‐analysis of prospective studies. Eur J Epidemiol. 2018;33:711‐722. 29417316 10.1007/s10654-017-0353-9 PMC6061127

[158] Arslan E , Yiginer O , Yavasoglu I , Ozcelik F , Kardesoglu E , Nalbant S . Effect of uncomplicated obesity on QT interval in young men. Pol Arch Med Wewn. 2010;120:209‐213. 20567204

[159] Delhey L , Jin J , Thapa S , Delongchamp R , Faramawi MF . The association of metabolic syndrome and QRS|T angle in US adults (NHANES III). Ann Noninvasive Electrocardiol. 2020;25:e12678. 31361074 10.1111/anec.12678 PMC7358789

[160] Oehler A , Feldman T , Henrikson CA , Tereshchenko LG . QRS‐T angle: a review. Ann Noninvasive Electrocardiol. 2014;19:534‐542. 25201032 10.1111/anec.12206 PMC4237708

[161] Ambrose JA , Singh M . Pathophysiology of coronary artery disease leading to acute coronary syndromes. F1000Prime Rep. 2015;7:8. 10.12703/P7-08 PMC4311268 25705391

[162] Bandeali S , Farmer J . High‐density lipoprotein and atherosclerosis: the role of antioxidant activity. Curr Atheroscler Rep. 2012;14:101‐107. 22441969 10.1007/s11883-012-0235-2

[163] Xepapadaki E , Zvintzou E , Kalogeropoulou C , Filou S , Kypreos KE . Tauhe antioxidant function of HDL in atherosclerosis. Angiology. 2020;71:112‐121. 31185723 10.1177/0003319719854609

[164] Hosadurg N , Bogle BM , Joodi G , et al. Lipid profiles in out‐of‐hospital sudden unexpected death. Mayo Clin Proc Innov Qual Outcomes. 2018;2:257‐266. 30225459 10.1016/j.mayocpiqo.2018.06.010 PMC6132208

[165] Kunutsor SK , Zaccardi F , Karppi J , Kurl S , Laukkanen JA . Is high serum LDL/HDL cholesterol ratio an emerging risk factor for sudden cardiac death? findings from the KIHD study. J Atheroscler Thromb. 2017;24:600‐608. 27784848 10.5551/jat.37184 PMC5453685

[166] Schillaci G , Vaudo G , Reboldi G , et al. High‐density lipoprotein cholesterol and left ventricular hypertrophy in essential hypertension. J Hypertens. 2001;19:2265‐2270. 11725172 10.1097/00004872-200112000-00021

[167] Horio T , Miyazato J , Kamide K , Takiuchi S , Kawano Y . Influence of low high‐density lipoprotein cholesterol on left ventricular hypertrophy and diastolic function in essential hypertension. Am J Hypertens. 2003;16:938‐944. 14573332 10.1016/s0895-7061(03)01015-x

[168] Talayero BG , Sacks FM . The role of triglycerides in atherosclerosis. Curr Cardiol Rep. 2011;13:544‐552. 21968696 10.1007/s11886-011-0220-3 PMC3234107

[169] Peng J , Luo F , Ruan G , Peng R , Li X . Hypertriglyceridemia and atherosclerosis. Lipids Health Dis. 2017;16:233. 29212549 10.1186/s12944-017-0625-0 PMC5719571

[170] Iso H , Naito Y , Sato S , et al. Serum triglycerides and risk of coronary heart disease among Japanese men and women. Am J Epidemiol. 2001;153:490‐499. 11226981 10.1093/aje/153.5.490

[171] Lemieux I , Poirier P , Bergeron J , et al. Hypertriglyceridemic waist: a useful screening phenotype in preventive cardiology? Can J Cardiol. 2007;23:23B‐31B. 10.1016/s0828-282x(07)71007-3 PMC2794461 17932584

[172] Wang Q , Chair SY , Wong EM , Taylor‐Piliae RE . Metabolic syndrome knowledge among adults with cardiometabolic risk factors: a cross‐sectional study. Int J Environ Res Public Health. 2019;16(1):159. 10.3390/ijerph16010159 PMC6338970 30626137

[173] Tseng ZH , Olgin JE , Vittinghoff E , et al. Prospective countywide surveillance and autopsy characterization of sudden cardiac death: POST SCD study. Circulation. 2018;137:2689‐2700. 29915095 10.1161/CIRCULATIONAHA.117.033427 PMC6013842

[174] Basso C , Carturan E , Pilichou K , Rizzo S , Corrado D , Thiene G . Sudden cardiac death with normal heart: molecular autopsy. Cardiovasc Pathol. 2010;19:321‐325. 20381381 10.1016/j.carpath.2010.02.003

[175] Turakhia M , Tseng ZH . Sudden cardiac death: epidemiology, mechanisms, and therapy. Curr Probl Cardiol. 2007;32:501‐546. 17723906 10.1016/j.cpcardiol.2007.05.002

[176] Basso C , Aguilera B , Banner J , et al. Guidelines for autopsy investigation of sudden cardiac death: 2017 update from the Association for European Cardiovascular Pathology. Virchows Arch. 2017;471:691‐705. 28889247 10.1007/s00428-017-2221-0 PMC5711979

[177] Leitner DR , Fruhbeck G , Yumuk V , et al. Obesity and type 2 diabetes: two diseases with a need for combined treatment strategies ‐ EASO can lead the way. Obes Facts. 2017;10:483‐492. 29020674 10.1159/000480525 PMC5741209

[178] Al‐Daydamony MM , El‐Tahlawi M . What is the effect of metabolic syndrome without hypertension on left ventricular hypertrophy? Echocardiography. 2016;33:1284‐1289. 27109543 10.1111/echo.13247

[179] Laukkanen JA , Khan H , Kurl S , et al. Left ventricular mass and the risk of sudden cardiac death: a population‐based study. J Am Heart Assoc. 2014;3:e001285. 25376188 10.1161/JAHA.114.001285 PMC4338721

[180] Kannankeril PJ , Goldberger JJ . Parasympathetic effects on cardiac electrophysiology during exercise and recovery. Am J Physiol Heart Circ Physiol. 2002;282:H2091‐H2098. 12003816 10.1152/ajpheart.00825.2001

[181] Kannankeril PJ , Le FK , Kadish AH , Goldberger JJ . Parasympathetic effects on heart rate recovery after exercise. J Investig Med. 2004;52:394‐401. 10.1136/jim-52-06-34 15612453

[182] Galetta F , Franzoni F , Fallahi P , et al. Effect of telmisartan on QT interval variability and autonomic control in hypertensive patients with left ventricular hypertrophy. Biomed Pharmacother. 2010;64:516‐520. 20044234 10.1016/j.biopha.2009.09.016

[183] Stanley J , Peake JM , Buchheit M . Cardiac parasympathetic reactivation following exercise: implications for training prescription. Sports Med. 2013;43:1259‐1277. 23912805 10.1007/s40279-013-0083-4

[184] Christensen NJ , Galbo H . Sympathetic nervous activity during exercise. Annu Rev Physiol. 1983;45:139‐153. 6342511 10.1146/annurev.ph.45.030183.001035

[185] Siscovick DS , Weiss NS , Fletcher RH , Lasky T . The incidence of primary cardiac arrest during vigorous exercise. N Engl J Med. 1984;311:874‐877. 6472399 10.1056/NEJM198410043111402

[186] Strasser B . Physical activity in obesity and metabolic syndrome. Ann NY Acad Sci. 2013;1281:141‐159. 23167451 10.1111/j.1749-6632.2012.06785.x PMC3715111

[187] Lakka TA , Laaksonen DE . Physical activity in prevention and treatment of the metabolic syndrome. Appl Physiol Nutr Metab. 2007;32:76‐88. 17332786 10.1139/h06-113

